# Use of Individually Designed CAD/CAM Suprastructures for Dental Reconstruction in Patients with Cleft Lip and Palate

**DOI:** 10.3390/dj11090212

**Published:** 2023-09-11

**Authors:** Pilvi Mäntynen, Marisa Laurila, Tommi Strausz, Jari Mauno, Junnu Leikola, Juho Suojanen

**Affiliations:** 1 Päijät-Häme Joint Authority for Health and Wellbeing, Department of Oral and Maxillofacial Surgery, Lahti Central Hospital, 15850 Lahti, Finland; marisa.laurila@paijatha.fi (M.L.); juho.suojanen@helsinki.fi (J.S.); 2 Clinicum, Faculty of Medicine, University of Helsinki, 00014 Helsinki, Finland; 3 Institute for Molecular Medicine Finland, Helsinki Institute of Life Science, University of Helsinki, 00014 Helsinki, Finland; 4 Cleft Palate and Craniofacial Centre, Department of Plastic Surgery, HUS Helsinki University Hospital and University of Helsinki, 00029 Helsinki, Finland; jari.mauno@hus.fi (J.M.); junnu.leikola@hus.fi (J.L.)

**Keywords:** CAD/CAM suprastructure, prosthetic reconstruction, implant-supported, cleft lip and palate, cleft lip, cleft alveolus, fixed prosthodontics, telescopic suprastructure, Atlantis, Createch

## Abstract

This patient series reports the outcomes of CAD/CAM prosthetic reconstructions in patients with cleft lip and palate (n = 9, aged 27 to 76) who have experienced significant failure with conventional restorative and fixed prosthodontic treatments. The objective of the protocol is to establish a functional and patient-friendly prosthetic structure for individuals with unilateral/bilateral cleft lip and palate (UCLP/BCLP) while minimising the requirement for specialised follow-up care in the cleft unit. The study data were obtained from a retrospective cohort at Helsinki University Hospital. Prosthetic reconstructions were performed using CAD/CAM bar structures by the Atlantis 2in1 system or Createch removable telescope structures, supported by four to eight maxillary dental implants. Out of the nine patients, seven experienced no complications. One prosthesis fracture occurred after 16 months due to a design error in the original framework, and one patient experienced failure of osseointegration in a dental fixture (specifically, one fixture out of the eight maxillary implants in this patient). In total, 56 implants were successfully placed. The maxillary dentition of elderly patients with cleft lip and palate often poses challenges due to periodontal and reconstructive issues. An implant-supported CAD/CAM bar with a removable telescope suprastructure offers an easily maintained and functional solution for dental rehabilitation.

## 1. Introduction

Clefts of the lip, alveolar ridge, and palate are the most common serious congenital anomalies to affect the orofacial region [1]. The incidence of cleft lip with or without cleft palate (CL±P) is approximately 1 in 1000 live births [2]. A cleft of the alveolus particularly affects the growth of the upper jaw and the development of primary and permanent teeth [1], which can occur through iatrogenic, functional, or intrinsic mechanisms [3]. Regardless of whether the cleft lip and palate are unilateral or bilateral, there is a high prevalence of dental anomalies associated with orofacial clefts [1]. However, patients with bilateral cleft lip and palate (BCLP) are reported to be affected more [4]. Missing teeth are a common problem in patients with cleft lip and palate (CLP), who may experience partial anodontia [5,6], and supernumerary teeth may also be present [6]. The dentition in the lower jaw is more often unaffected, although missing lower teeth are more common in CLP patients. A study by Tan et al. examined dental maturation in patients with UCLP and compared the findings to those from children without cleft. They found that on the cleft side, the teeth of the maxilla had a significantly higher risk of delayed development compared to the teeth on the non-cleft side of the mandible (RR = 2.39, 95% CI 1.11–5.17; *p* = 0.027) [7].

Orthodontic treatment and corrective jaw surgery are often needed due to malocclusions. The growth of the maxilla in patients with CLP differs from skeletal Class I, but it does exhibit a similarity to the growth pattern observed in skeletal Class III. The evident slowdown in maxillary growth among those with cleft lip and palate highlights the importance of implementing early-stage treatment approaches like maxillary protraction and expansion [8]. A severe cleft is associated with unfavourable maxillary forward growth [9]. Additionally, poor growth of the maxilla can result from iatrogenic trauma during the primary operative closure [10]. Closing the palate in a single stage at the age of 1 or earlier interferes with maxillary growth more than a two-step palatoplasty [11].

Patients with CLP require surgical interventions at various life stages. Variations in surgical protocols exist among different medical centres [12]. At HUH (Helsinki University Hospital), the initial lip surgery typically takes place around 3 to 4 months after birth, while palatoplasty is most often performed between 9 and 12 months of age. Additionally, secondary bone graft procedures, such as utilising bone grafts from the iliac crest, are performed on patients aged 10 to 12 years, during the late mixed dentition phase. The bone grafting process includes surgical procedures such as closing fistulas, supporting the alar base, and providing alveolar support. The presence of a bilateral cleft can potentially lead to issues related to blood supply. Therefore, a two-stage surgical approach should be considered [13]. In our unit, for patients with severe maxillary hypoplasia, an early advancing maxillary osteotomy is performed by the age of 12 to 14, followed by a finalising osteotomy after growth. 

The alveolar bone on the buccal and palatal sides of teeth anterior to the cleft is significantly thinner compared to teeth unaffected by a cleft [14]. The lack of alveolar bone results in poor periodontal support for teeth adjacent to the cleft margins, increasing the risk of early tooth loss. Patients presenting with clefts exhibited a heightened susceptibility to inferior periodontal outcomes. Subsequent subgroup analysis unveiled compromised periodontal metrics within cleft-affected sites, marking a significant statistical contrast in terms of gingival index, clinical attachment level, and plaque index [15]. This indicates that the presence of orofacial clefts may confer a proclivity towards compromised periodontal health when juxtaposed with normative controls. The presence of cleft lip and/or palate also has a negative impact on oral hygiene and the levels of periodontopathogens in the oral biofilm. Individuals with CL/P are more likely to exhibit higher plaque accumulation and gingival inflammation [16,17]. 

**Figure 1:**
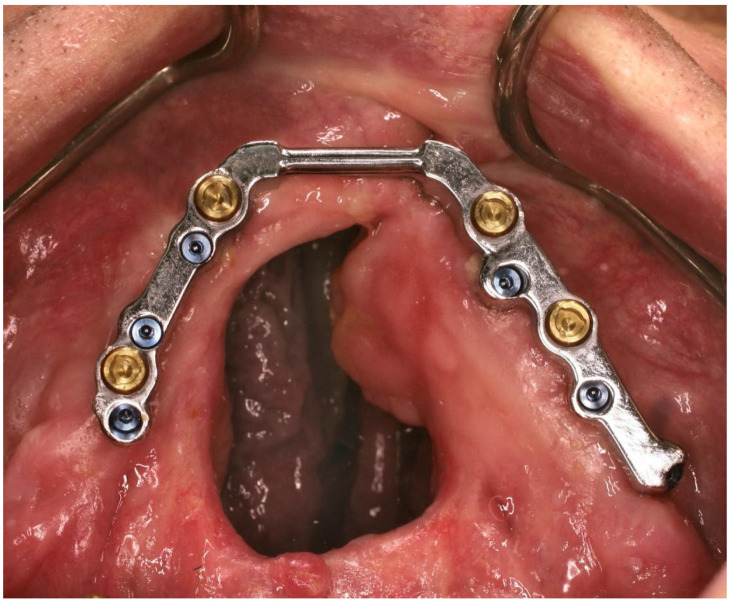
The primary suprastructure with Locator attachments is fixed to the abutments. A residual oronasal fistula in the palate can complicate treatment with conventional restorative and fixed prosthodontics. Patient no. 8.

Some of the patients also have residual oronasal fistulas in the palate, which can complicate treatment (Figure 1). The Veau classification is a commonly used system for characterising the severity of cleft palate. Patients classified as Veau IV, with bilateral cleft lip and palate, have the highest risk of developing fistulas (OR = 10.582; *p* = 0.004) [18]. Additionally, a higher number of complications has been reported when performing bone grafting followed by dental implant insertion in patients with a maxillary alveolar cleft defect compared to those with other cleft defects. This has been attributed to the presence of oronasal fistulas and scarring of soft tissues [19]. 

Edentulism is a prevalent issue worldwide, particularly among the elderly population. Factors contributing to the necessity of tooth extraction include dental caries, periodontitis, and socioeconomic status. Complete dentures are regarded as a viable treatment modality for the oral rehabilitation of the edentulous maxilla [20]. Other options for oral prosthetic rehabilitation of edentulous maxilla are implant-supported fixed prostheses or implant-retained removable prostheses. Due to possible deficiencies of hard and soft tissues and abnormalities in the oral cavity in patients with CLP, prosthetic oral rehabilitation with conventional prostheses may not be the preferred option [21]. Implant-supported fixed prostheses are considered superior to implant-retained removable prostheses in terms of implant survival rates [22]. However, in our study, factors such as unfavourable maxilla–mandibular bone relations and lack of alveolar bone in the anterior region of the maxilla led to the decision to use implant-retained removable prostheses.

The aim of this study is to implement a durable and patient-friendly prosthetic structure that is easy to clean, thereby reducing the demand and frequency of maintenance treatments with specialists. The prosthetic reconstructions in this study were implemented as CAD/CAM bar structures, specifically using the Atlantis 2in1 system or Createch removable telescopic structures. This approach allowed us to achieve a structure that can be implemented with reasonable operations and risks. When planning the treatment, it is crucial to consider the unique characteristics of this challenging patient group, such as the lack of alveolar bone and unfavourable maxilla–mandibular bone relations. The objective of this study is to analyse the challenges and outcomes of treating UCLP/BCLP patients who have edentulous or partly edentulous maxilla with CAD/CAM suprastructures. It is worth remembering that the advantages of a multidisciplinary team strategy for the comprehensive care of patients with CLP and their families must be highlighted [23].

## 2. Materials and Methods

The study data were derived from a retrospective cohort of cleft lip and palate patients who were treated at the Helsinki University Hospital (HUH). These patients received treatment at the HUH Cleft Palate and Craniofacial Centre between the years 2010 and 2019. The centre serves as a national referral centre for cleft patients, providing both primary and secondary reconstructions throughout Finland. The research plan was approved by the regional board for research (ref: HUS / 576/2019). This case series includes prosthetic reconstructions of cleft lip and palate patients (n = 9, aged 27 to 76) who experienced severe failure of conventional restorative and fixed prosthodontics for various reasons. In cases where extraction of residual dentition in the upper jaw was necessary, patients with edentulous maxilla were treated with overdentures supported by four to eight dental implants. The treatment took place at the Cleft Palate and Craniofacial Centre HUSUKE (Helsinki University Hospital, Helsinki, Finland) from 2012 to 2019. The patients in this cohort had previously undergone surgical operations for cleft palate and lip and were hesitant to undergo extensive bone reconstructions. Treatment options were discussed with the patients prior to initiating treatment, and their expectations were to have a stable and durable bridge-like prosthetic solution.

Non-smoking was not an absolute requirement before the treatment, but patients were informed about the harmful impacts of it. The goal was to minimise smoking to a maximum of 5 cigarettes per day. When bone graft procedures were performed or implants were installed, patients were required to be either non-smokers or have a low level of smoking.

**Figure 2:**
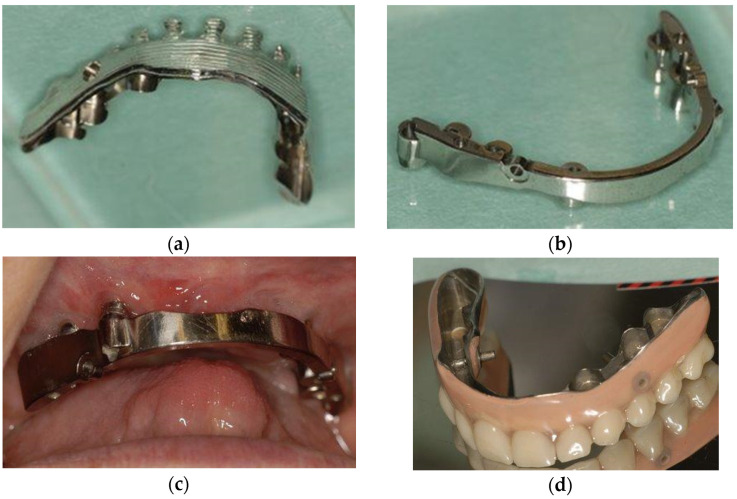
CAD/CAM prosthetic structure. The Atlantis 2in1 structure combines bar and box constructs with interlocking pins. (**a**) The framework of the secondary suprastructure (without custom teeth and denture resin); (**b**) the primary suprastructure that will be fixed to patients’ abutments; (**c**) the primary suprastructure fixed to the patient’s abutments; and (**d**) the finalised secondary suprastructure, with the custom teeth and denture resin manufactured on the titanium framework.

Prosthetic reconstructions have been implemented as CAD/CAM bar structures using the Atlantis 2in1 system (Figure 2) (Dentsply Sirona, Charlotte, NC, USA), or Createch removable telescopic structures (Createch Medical S.L.; Mendaro, Spain). The Atlantis 2in1 system consists of two suprastructures: a primary suprastructure that is fixed and anchored to implants and a hybrid secondary suprastructure that is removable and attached to the primary suprastructure. This attachment is achieved through friction, utilising a milling degree of four degrees, and additional elements, such as precision attachments incorporated in the bar structure. The removable hybrid secondary suprastructure is layered with custom teeth and denture resin. The Createch removable telescopic structure is similar to the Atlantis 2in1 system. It also consists of two structures attached to each other through friction with additional retention elements incorporated in the bar structures. Both structures feature a titanium framework in primary and secondary suprastructures. The precision attachments used in this case series were MK1 sliding bolt precision attachments, CEKA attachments (Ceka Revax M2 Axial Titanax Bonding, Ceka Preci-Line, Alphadent Nv, Waregem, Belgium) or Locator attachments (Zest Dental Solutions, Carlsbad, CA, USA).

The treatment began with consultation appointments in which patients were examined and their situations evaluated. Following the examination, previous prosthetic reconstructions were dismantled, and infections treated if necessary. Temporary removable dentures were then manufactured. Planning for bone reconstructions and sinus lift procedures commenced at this stage. Extraction of affected maxillary teeth followed the planning stage, and a healing period of six months was observed. Seven patients underwent sinus lift procedures, while eight patients received minor alveolar bone reconstructions using autogenous bone chips or Bio-Oss Collagen (Geistlich Pharma AG, Wolhusen, Switzerland), or an iliac crest bone block graft (Table 1). Larger sandwich-type bone reconstructions were not employed. 

For 3D implant planning, cone beam computed tomography (CBCT) scans with tooth setup were taken for each patient. The tooth setups were created by the prosthodontist, who was also responsible for the entire treatment of each patient. Implant planning was then performed virtually by author JM with Romexis software (Planmeca Oy, Helsinki, Finland). At this stage, the final prosthetic treatment plan was developed, considering factors such as patient preferences, available vertical space for prosthetic structures, lip support, and occlusion. After a healing period of six months, the patient underwent implant surgery based on the treatment plan. The dental implants used included Ankylos (Dentsply Sirona, Charlotte, NC, USA), AnyRidge (MegaGen, Daegu, Republic of Korea), XiVE (Dentsply Sirona, Charlotte, NC, USA), and Straumann BL (Straumann Holding AG, Basel, Switzerland). The number of implants varied from four to eight, depending on the bone level and mucosal tissue.

After an osseointegration period of three to six months, the implants were uncovered in second-stage implant surgery, and the final bridge or bar abutments were placed. The gingival height of the final abutments was adjusted to match the soft tissue margin, allowing for easy and controllable placement of the suprastructure. The clinical procedure for placing the Atlantis 2in1 prosthetic reconstruction and Createch removable telescopic structure was similar in both prosthetic systems. Firstly, definitive impressions were taken using a custom open tray technique at the abutment level with polyether impression material (Impregum Penta Soft, 3M Oral Care, St. Paul, MN, USA). For the final precision impression, impression copings were rigidly splinted together using an individual metal frame and autopolymerising acrylic resin (Palavit G, Kulzer, Hanau, Germany). The splinted structure was then embedded into the impression material (Impregum Penta Soft) using individual impression trays. The individual metal frames employed in precision impression were crafted from cobalt–chrome alloy and were meticulously fabricated by a dental technician. If necessary, the trimming was performed in the final impression using Kerr Impression Compound green (Kerr, Uxbridge, Great Britain). The casts were mounted, and the processed denture base with wax occlusion rim was fabricated by a dental technician. Then, the framework was made and fitted to the patients. The processed denture base was then evaluated at the abutment level, and interocclusal registration was performed using a wax occlusion rim. Then, the casts were mounted on an articulator (Artex articulator, Amann Girrbach AG, Koblach, Austria) using an interocclusal bite registration. At the next clinical appointment, the denture base with definitive tooth arrangement, as well as the contouring of the denture base and aesthetics, was evaluated. At this stage, the definitive tooth arrangement was marked with the intended positions of the precise attachments and then delivered, along with the definitive cast, to the manufacturer of the suprastructures for scanning. The primary suprastructure and the framework of the secondary suprastructure were planned and manufactured using CAD/CAM technology. The design and cleanability of the framework were approved in a digital format prior to milling. The precision attachments were chosen according to the patient’s needs and motor skills. The sliding bolt attachment is designed to mechanically secure the suprastructure, preventing unnecessary structural erosion caused by microscopic movements during biting. The bar structure was combined with three to four Ceka or Locator attachments, symmetrically positioned to provide retention for the night-time splint. Ceka attachments were the preferred choice due to their compact size and easily convertible components. These interlocking attachments were not activated in the secondary suprastructure if the patient was using the MK1 sliding bolt. In cases where a patient’s manual dexterity decreases with age or due to other factors, it is possible to reduce the frictional attachment of the structure, remove the MK1 attachments, and activate Ceka or Locator attachments. Before delivering the final prosthetic structures, the passive fit of the framework of the primary suprastructure was evaluated using the Sheffield test [24]. The fit between the primary and secondary suprastructures was also estimated and confirmed on patients, and the final definitive tooth arrangement was evaluated and trimmed if necessary. It was also ensured that the cleaning of the structures was possible for the patients to maintain proper oral health. Most of the overdentures were horseshoe-shaped, except for one that had palatal coverage to address a large oronasal fistula (Figure 1).

The prosthetic structures were completed in the dental laboratory, delivered to the hospital, and evaluated during patients’ appointments. The primary bar was fixed to the abutments with the specified torque for each patient, and the secondary suprastructures were fitted and adjusted accordingly. Additionally, occlusal splints were custom-made for the patients to protect the primary bar structures during night-time. The occlusal splints were manufactured to match the vertical height of the prosthetic structures. 

The patients were provided with hygiene instructions and were taught how to use the removable suprastructures. Maintenance treatment was clinically administered by the patient’s regular general dentists or dental hygienists outside of specialised medical care. The prosthetic structures were supervised within the Cleft Unit, with follow-up appointments scheduled at one month, six months, and twelve months, followed by annual appointments until the fifth year. Beyond that, appointments were only arranged in the event of structural issues. In such instances, patients were able to directly schedule an appointment with the Cleft Unit by contacting them or providing a cover letter from their dentist.

Appointments at the cleft unit involved the evaluating of the clinical status of dental implants, prosthetic structures, and occlusion. Panoramic tomography was performed during the 12-month check-up and, in many cases, during the 4- or 5-year check-up as well. In Finland, there is an emphasis on reducing the routine use of X-rays, and they are conducted only when clinically warranted, such as in cases of bleeding on probing or deep periodontal pockets.

## 3. Results

The patient demographics can be seen in detail in Table 2. The patient series consists of prosthetic reconstructions of cleft lip and palate patients (n = 9, aged 27 to 76, five male and four female) with a history of severe failure of conventional restorative and fixed prosthodontics and functional occlusion for different reasons: secondary caries in dental bridge (three patients), secondary caries in dental crowns (one patient), and periodontitis (four patients). One of the patients had edentulous jaws when starting this treatment period; the reason for this was unknown. Three of the nine patients had bilateral cleft lip and palate (BCLP), and six of them were unilateral (UCLP).

**Figure 3:**
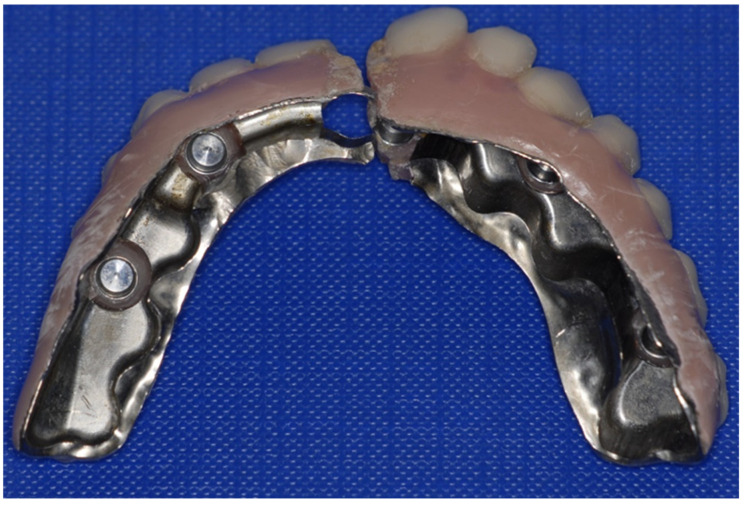
Prosthetic failure: A prosthesis fracture occurred after 16 months due to a design error in the original framework (Patient no. 1). The load-bearing connector area had been designed too thin/narrow, as determined in a retrospective analysis.

Seven patients out of the nine had no complications (Table 3). One prosthesis fracture occurred at 16 months due to a design error in the original framework (Createch), where the load-bearing connector area designed was too thin/narrow (Figure 3). One patient experienced a failure of osseointegration in one out of eight implants, but the prosthodontic suprastructure could be designed without issues. The survival rate for the cohort in primary osseointegration was 55 out of 56 (98%). The mean follow-up period of the patients was 37 months before being referred to their regular dental practitioners.

## 4. Discussion

The aim of the study was to analyse the challenges and outcomes of the treatment of UCLP/BCLP patients with edentulous or partly edentulous maxilla using dental implant-supported CAD/CAM bar suprastructures. 

Patients with cleft lip and palate often require complex dental restorations. Various treatment options are available for prosthetic oral rehabilitation in this patient group, including fixed partial dentures, removable dentures, and precision prostheses [25]. Additionally, several attachment systems have been suggested for implant-supported overdentures (IODs), such as ball attachments, Locator-type attachments, bars, and magnets [26], as well as external resilient attachments and Hader clips [21]. When deciding on the prosthetic structures, careful consideration needs to be given to the patient’s motor skills for implementing oral hygiene and managing the prosthetic structure [27], as well as their personal preferences and anatomical factors such as lip support, possible soft and hard tissue deficiencies, and the occlusion [28]. The costs of the prosthetic treatment, as well as the costs of the maintenance of oral hygiene [27] and possible repairs of structures, are important considerations. The impact of at-home oral hygiene practices on the maintenance of oral health, encompassing both natural teeth and dental implants, is vital. In our study, it was observed that when a patient has a bar structure that is easily maintainable, there is a notable decrease in the occurrence of gum-related issues, such as gingivitis.

Patients with CLP often exhibit restricting scars in the mucosal and underlying tissues, bone, and soft tissue deficits, as well as a skeletal Class III malocclusion [8]. Additionally, this group of patients may have abnormalities in the oral cavity, which can result in inadequate border seals of conventional prostheses [29]. The existing literature provides limited evidence regarding the advantages of IODs in the maxilla. However, substantial evidence concerning IODs in the mandible demonstrates their ability to augment patient satisfaction, amplify bite force, and enhance chewing capability [30], while also providing better retention and stability compared to conventional prostheses [31]. Hence, implant-retained prostheses may be a favourable treatment option for this patient group, especially among patients with CLP, despite the limited available literature on implant-retained dentures in the maxilla.

Due to the potential instability of the left and right sides of maxillary arches in patients with CLP, it is crucial to have a stable and rigid framework for the overdenture. In addition, patients with CLP often exhibit poor alveolar bone quality in the anterior maxilla, with a significantly thinner alveolar bone on the buccal and palatal sides of the teeth anterior to the cleft compared to non-cleft teeth. Furthermore, reduced alveolar bone height is more commonly observed on the cleft side compared to the control side in UCLP patients [32]. In our study, the dental implants were positioned more posteriorly, which also necessitates a rigid prosthetic framework. Considering these factors and aiming to provide patients with easily maintainable prosthetic structures, implant-retained hybrid removable overdentures were chosen. 

Zanolla et al. studied the success rates of IODs and IFDs in patients with CLP. The study reported an implant survival rate of 88.46%, with IODs having a survival rate of 81.5% and IFDs having a survival rate of 95.39% [33]. However, it should be noted that the study by Zanolla et al. included implants inserted in both the maxilla and mandibula. In a systematic review by Wermker et al., the mean implant success rate in patients with CLP was found to be 88.6%. However, the available evidence is limited due to a lack of sufficient clinical studies [34]. Comparatively, implant survival rates in IODs and IFDs in patients with CLP are lower than those in non-cleft patients [35]. It has been recommended in the literature that a minimum of four to six splinted implants are needed to support a maxillary overdenture without palatal coverage [36]. In our study, all patients had four or more implant fixtures, with four patients having seven or eight implant fixtures. Having an adequate number of implant fixtures can facilitate the repair process of the prosthetic structure in the event of an implant complication. 

Another treatment option for edentulous patients with a history of CLP is, for example, zygomatic implant-supported prosthodontic rehabilitation, as studied by Leven et al. in their clinical report [37]. They reported seven edentulous patients treated successfully with zygomatic implants. However, only a few reports on zygomatic implant-supported prosthetic rehabilitation in cleft patients exist. M. Mommaerts reports that for patients with severe bone atrophy, additively manufactured sub-periosteal implants provide functional restoration with only one surgical appointment [38]. However, there are no reports of this kind of treatment in patients with CLP. 

In our study, CAD/CAM-fabricated milled bars and suprastructures were used for prosthetic rehabilitation. Previously, bars were cast, which often resulted in misfits of the bar structures. The use of computer-aided milling procedures has helped address this problem. However, achieving a passive fit of the bars to fixtures remains a challenge [39]. One approach to prevent issues with passive fit is to rigidly splint impression copings together using an individual metal frame and autopolymerising acrylic resin. Instead of taking analogue definitive impressions, intraoral scanning can be considered. Intraoral digital scanning has become common, particularly in fixed prosthetics, and it can also be utilised for the type of prosthetic structures used in our study [40]. However, the ITI Consensus Report recommends caution in using digital impressions for large interimplant spans and digital implant impressions of edentulous jaws in routine clinical practice [41]. Additionally, it is important to consider whether intraoral scanners can accurately capture the soft tissues of patients with CLP, especially when there may be a fistula present in the palate and scarring in the soft tissues.

In our study, nine patients with CLP and edentulous maxilla were examined and provided with implant-retained hybrid removable overdentures, and treatments indicated the potential of this type of prosthetic structure. However, it is important to note that one framework fracture occurred due to a design error, emphasising the significance of careful prosthetic structure design. Also, the follow-up time varied from twelve months to one hundred and eighteen months, with only five patients being monitored over a three-year period and two patients over an eight-year period. Therefore, the available data are insufficient to draw conclusions on the success rates over a long period of time. As a future perspective, a randomised clinical trial should be performed to evaluate the follow-up of these kinds of restorations. Additionally, this consideration should be incorporated into the study design.

Another limitation of this study is that it is a retrospective cohort study with a small number of patients. However, the HUH Cleft Unit provides its services nationally and the care is provided without patient fees for the prosthetic works. The treated patients are free to contact the unit if complications arise or if any problems with the prosthetic constructs occur. Therefore, it can be assumed that we would have been informed either by the patients themselves or their general dentists if significant complications had occurred. Undoubtedly, the difficulty in enrolling patients is a limitation. Also, the patient health record system has been altered in a way that the scanned anamnestic information is no longer accessible and cannot be reliably evaluated in a retrospective cohort.

Additionally, there are only a limited number of studies in the literature that investigate prosthetic structures similar to those used in our study. Despite the lack of evidence supporting the use of this prosthetic structure, it is found to be promising. Further research is necessary to gather information on the long-term survival and success rates.

## 5. Conclusions

The maxillary dentition of cleft lip and palate patients can pose challenges, particularly in elderly patients, due to periodontal and reconstructive issues. In comparison to the mandible, the maxillary teeth often exhibit a poorer condition. Alveolar hypoplasia and compromised soft tissues make dental rehabilitation with conventional fixed prosthodontics challenging. However, utilising a dental implant-supported CAD/CAM bar with removable telescope suprastructure offers a functional and easily maintainable solution for their dental rehabilitation.

## Figures and Tables

**Table 1:** Surgical interventions.

Patient No.	Bone Grafting	Sinus Lift
1	Autogenous bone chips	Hydrolift
2	None	Condensation
3	Iliac crest	None
4	BioOss	Hydrolift
5	Iliac crest	Lateral window
6	Iliac crest	None
7	None	None
8	Iliac crest	Lateral window
9	None	None

**Table 2:** Demographic data of the patients.

Patient No.	Sex	Diagnosis	Age (y)	Primary Reason for Treatment	Earlier Cleft and CMFS Operations	Opposing Dentition
1	M	BCLP	65	Secondary caries in dental bridge	Primary closure 4 mo, palatoplasty 2 y, Le Fort I 41 y.	Own teeth
2	F	BCLP	65	Secondary caries in dental bridge	Primary closure 6 y, naso- and rhinoplasty 10 y, palatoplasty 11 y, lip plasty 13 y, oronasal fistula closure 20 y.	Partial removable denture
3	M	UCLP	66	Periodontitis	Primary closure unknown. No cleft augmentation.	2in1
4	M	UCLP	61	Periodontitis	Primary closure 2 mo, palatoplasty 2.5 y, lip and rhinoplasty 6 y, lip plasty 7 y, fistula closure 15 y.	Own teeth
5	F	UCLP	54	Periodontitis	Primary closure unknown. Palatal closure 2 y.	Own teeth
6	F	UCLP	62	Secondary caries in dental crowns	Primary lip closure 4 mo, palatal closure 2 y, lip plasty 20 y.	Partial removable denture
7	F	UCLP	27	Periodontitis	Primary closure 3 mo, cleft augmentation 13 y.	Own teeth
8	M	BCLP	76	Edentulous jaws, reason unknown	Primary closure unknown as small child. No later operations.	Complete denture
9	M	UCLP	47	Secondary caries in dental bridge	Primary closure unknown. Le fort I osteotomy. Septoplasty.	Own teeth

Abbreviations: F, female; M, male; BCLP, bilateral cleft lip and palate; UCLP, unilateral cleft lip and palate; y, year; mo, month; CMFS, craniomaxillofacial surgery.

**Table 3:** Characteristics of study patients.

Patient No.	Oronasal Fistula	Number of Mx Implants	Type of Fixture	Prosthodontics	Complications	Follow-Up (mo)
1	Significant	6	AnyRidge	Createch	Prosthesis fracture 16 months	45
2	Significant	6	Ankylos	Createch	None	22
3	None	4	Ankylos	Createch	None	49
4	None	8	Ankylos	Isus/Atlantis	Failure of osseointegration (n = 1)	49
5	None	7	Ankylos	Createch	None	29
6	None	8	Ankylos	Isus/Atlantis	None	102
7	Minor	8	Straumann BL	Createch	None	13
8	Significant	4	Ankylos	Createch	None	12
9	Minor	5	Ankylos	Isus/Atlantis	None	12

Abbreviations: mx, maxilla.

## Data Availability

Single patient data are not available due to GDBR.
